# A Comprehensive Pan-Cancer Analysis of the Potential Biological Functions and Prognosis Values of RICTOR

**DOI:** 10.3390/genes14061280

**Published:** 2023-06-16

**Authors:** Ying Sun, Rui Li, Baoting Nong, Zhou Songyang, Xianren Wang, Wenbin Ma, Qin Zhou

**Affiliations:** 1 Department of Nephrology, The First Affiliated Hospital, Sun Yat-sen University, Guangzhou 510080, China; 2 MOE Key Laboratory of Gene Function and Regulation, Guangzhou Key Laboratory of Healthy Aging Research and SYSU-BCM Joint Research Center, School of Life Sciences, Sun Yat-sen University, Guangzhou 510275, China; 3 Breast Tumor Center, Sun Yat-sen Memorial Hospital, Sun Yat-sen University, Guangzhou 510120, China; 4 Department of Otolarygology, The First Affiliated Hospital, Sun Yat-sen University, Guangzhou 510080, China; wangxren@mail.sysu.edu.cn; 5 NHC Key Laboratory of Clinical Nephrology and Guangdong Provincial Key Laboratory of Nephrology, Sun Yat-Sen University, Guangzhou 510080, China

**Keywords:** Pan-cancer, mTORC2, RICTOR

## Abstract

The importance of the network defined by phosphatidylinositol-3-kinase (PI3K), AKT and mammalian target of rapamycin (mTOR) downstream of Receptor Tyrosine Kinase (RTK) has been recognized for many years. However, the central role of RICTOR (rapamycin-insensitive companion of mTOR) in this pathway has only recently come to light. The function of RICTOR in pan-cancer still needs to be systematically elucidated. In this study, we examined RICTOR’s molecular characteristics and clinical prognostic value by pan-cancer analysis. Our findings indicate that RICTOR was overexpressed in twelve cancer types, and a high RICTOR expression was linked to poor overall survival. Moreover, the CRISPR Achilles’ knockout analysis revealed that RICTOR was a critical gene for the survival of many tumor cells. Function analysis revealed that RICTOR-related genes were mainly involved in TOR signaling and cell growth. We further demonstrated that the RICTOR expression was significantly influenced by genetic alteration and DNA-methylation in multiple cancer types. Additionally, we found a positive relationship between RICTOR expression and the immune infiltration of macrophages and cancer-associated fibroblasts in Colon adenocarcinoma and Head and Neck squamous cell carcinoma. Finally, we validated the ability of RICTOR in sustaining tumor growth and invasion in the Hela cell line using cell-cycle analysis, the cell proliferation assay, and wound-healing assay. Our pan-cancer analysis highlights the critical role of RICTOR in tumor progression and its potential as a prognostic marker for various cancer types.

## 1. Introduction

mTORC2 is a protein complex that plays a crucial role in multiple biological processes in cells, such as cell proliferation, metabolic regulation, cell polarity, and cell apoptosis. It consists of mTOR, RICTOR (rapamycin insensitive companion of mTOR), SIN1 (stress-activated map kinase-interacting protein 1), mLST8 (mammalian lethal with SEC13 protein 8) and other regulatory subunits [1,2]. Studies have increasingly shown that mTORC2 can promote numerous types of cancer, such as prostate and breast cancers, making it a potential target for novel cancer therapies [3,4,5,6]. As a critical subunit of the mTORC2 complex, RICTOR regulates the activity of mTORC2 and its downstream signaling pathways by interacting with mTOR, mLST8, and other subunits [7]. Previous research has shown that the deletion of RICTOR can significantly delay the tumorigenesis of pancreatic cancer [8]. Additionally, RICTOR has been identified as the most frequently amplified gene in a cohort of metastatic, small, cell lung cancer (SCLC) patients (~14% of patients) and SCLC patients, with RICTOR amplification having a significantly decreased overall survival [9]. These results suggest that RICTOR plays a critical role in maintaining the survival of tumor cells and could be a promising target for cancer treatment.

Although there is growing evidence supporting the important role of RICTOR in the tumorigenesis of specific cancer types, its mechanism of action in cancer remains poorly understood. Additionally, there has been no comprehensive pan-cancer analysis of RICTOR to date. Therefore, the aim of this study was to comprehensively analyze the expression pattern, mutation status, methylation levels, prognostic value, and potential association between RICTOR expression and immune function in various cancers. From the results, we found that 29 types of cancer exhibited abnormal RICTOR gene expression, 10 types of cancer exhibited RICTOR mutation dysregulation, and 9 types of cancer exhibited methylation dysregulation. We further demonstrated that the RICTOR expression was significantly influenced by genetic alteration and DNA-methylation in multiple cancer types. These findings may provide new insights into the therapeutic targeting of RICTOR on different cancer types.

## 2. Materials and Methods

We obtained the mRNA expression profiles in tumor tissues and their corresponding normal tissues from The Cancer Genome Atlas (TCGA), Therapeutically Applicable Research to Generate Effective Treatments (TARGET) (https://www.cancer.gov/ccg/research/genome-sequencing/target, accessed on 15 February 2023), and the Genotype-Tissue Expression (GTEx) database (cancer list in Supplementary Table S1). The analysis of DNA methylation and protein expression level were performed by UALCAN [10] (http://ualcan.path.uab.edu/index.html, accessed on 15 February 2023). The TISIDB database [11] (http://cis.hku.hk/TISIDB/index.php, accessed on 15 February 2023) was employed to check the correlation between the RICTOR expression and molecular subtypes of cancer.

### 2.1. Survival Analysis

In order to investigate the potential impact of RICTOR expression levels on clinical outcomes, the cancer patients were divided into two groups, namely, high and low expression based on the median gene expression value. We then used Kaplan–Meier (KM) survival curves to assess the survival differences between the two patient groups with the PrognoScan website [12] (http://dna00.bio.kyutech.ac.jp/PrognoScan/, accessed on 18 February 2023). Using data from the TIDE website [13] (http://tide.dfci.harvard.edu, accessed on 19 February 2023), we also assessed the correlation between the methylation levels of RICTOR and the overall survival (OS) in pan-cancer data. To ascertain statistical significance, we established a threshold of 0.05 for the *p* value.

### 2.2. Genetic Alteration Analysis

From the cBioPortal platform [14] (https://www.cbioportal.org/, accessed on 23 February 2023), we obtained the mutation status of RICTOR. Additionally, survival analyses were performed to compare the survival outcomes of cancers with and without genetic alterations of RICTOR.

### 2.3. Protein–Protein Interaction Comprehensive Analysis

The online tool, Retrieval of Interacting Genes/Proteins (STRING) [15] (https:// string-preview.org, accessed on 25 February 2023), offers a vast collection of integrated and consolidated protein–protein interaction data. We used this tool to obtain the protein–protein interaction (PPI) network of RICTOR. Additionally, we utilized the function-predicting website Genemania [16] (http://genemania.org/, accessed on 25 February 2023) to investigate the interaction network of RICTOR. For both websites, we selected the top 10 genes that showed correlation with RICTOR for further Gene Ontology (GO) and Kyoto Encyclopedia of Genes and Genomes (KEGG) enrichment analysis. These analyses were performed using the R package “clusterProfiler” [17], which helped us determine the biological and molecular functions of the interaction genes. The GEPIA2 [18] (http://gepia2.cancer-pku.cn/#index, accessed on 25 February 2023) and TIMER2.0 [19] (http://timer.cistrome.org/, accessed on 25 February 2023) were used to perform expression correlation analysis between the interaction genes in pan-cancer data.

### 2.4. RICTOR Gene Essential Analysis by the CRISPR Dependency Scores

Different types of cancers have essential genes that are necessary for their survival. The Cancer Dependency Map project [20] (DepMap, www.depmap.org, accessed on 27 February 2023) aims to identify these gene dependencies by analyzing hundreds of cancer cell lines. We utilized DepMap to investigate the impact of RICTOR on the growth and survival of cancer cells. DepMap (Public 22Q4 + Score, Chronos) provides a comprehensive library of human genes that have been knocked down through RNAi or knocked out via CRISPR in various human cancer cell lines that represent different cancer types. The Chronos dependency scores indicate the probability of each cell line being dependent on the particular gene. Essential genes are denoted by negative values, with more negative values indicating greater importance. A score of 0 indicates a non-essential gene, while scores below −0.5 and −1 represent sensitive cell lines (most cells are depleted) and lethal cell lines, respectively.

### 2.5. Correlation between RICTOR Expression and Cancer Stemness

The Pearson correlation between RICTOR expression and cancer stemness was analyzed by the Sangerbox platform. The stemness was from the RNA-based stemness scores calculated by the stemness group [20].

### 2.6. Immune Analysis Tools

The “Immune Association” module of TIMER2 website (http://timer.cistrome.org/, accessed on 2 March 2023) was used to investigate the relationship between RICTOR expression and the infiltration of immune cells, such as cancer-associated fibroblasts (CAFs) and macrophages. Six state-of-the-art algorithms, including TIMER [21], xCell [22], MCP-counter [23], CIBERSORT [24], EPIC [25] and quanTIseq [26] were used to investigate the correlation between RICTOR expression levels and immune cell types associated with pan-cancer.

The ESTIMATE package was employed to estimate the presence of interstitial and immune cells in malignant tumor tissues using gene expression data [27]. It utilizes single-sample GSEA (ssGSEA), an extension of Gene Set Enrichment Analysis (GSEA), to generate three scores: stromal score, which measures the presence of stroma in tumor tissue; immune score, which reflects the infiltration of immune cells in tumor tissue; and estimated score, which infers tumor purity. The Sangerbox platform was employed to calculate the correlation between RICTOR expression levels and the three scores.

Furthermore, we investigated the correlation between RICTOR expression and immune-related genes, including chemokines, chemokine receptors, MHC genes, immunostimulator, immunoinhibitor, and immune checkpoint genes (both inhibitory and stimulatory) [27,28]. Pearson correlation analysis was performed to assess the association between RICTOR expression and these immune-related genes. These analyses can be achieved through the Sangerbox platform.

### 2.7. Cell Cycle Analysis

Cell pellet were harvested and suspended in 70% ethanol at 4 °C for 2 h. After centrifugation at 300× *g* for 5 min, the supernatant was aspirated. The cell pellet was resuspended in PBS and centrifuged at 300× *g* for 5 min, and the supernatant was discarded. The fixed cell pellet was then resuspended in PI staining solution containing RNase A and incubated for 15 min while being protected from direct light exposure. Finally, the cells were analyzed using flow cytometry.

### 2.8. Cell Growth Assay

Cell counting kit-8 (Selleck Chemicals, Houston, Texas, USA) was used in accordance with the manufacturer’s instructions. Hela cells were seeded in 96-well plates (1000 cells/well) and cultured at 37 °C in an incubator. A total of 10 μL of CCK8 solution was added to each well after 24, 48, 72, 96 and 120 h, respectively, and incubated for 2 h. Relative cell density was determined at a wavelength of 450 nm using the Biotek Synergy HTX (BioTek Instruments, VT, USA).

### 2.9. Cell Wound-Healing Assay

Hela cells were seeded into six-well plates at a density that allows cells to grow to confluency within 1–2 days. Once cells are confluent, we make a scratch in the monolayer with a pipette tip, creating a “wound”; wash cells twice with PBS to remove any cellular debris, and add fresh medium to cells; then take images of the cells at 0 and 48 h after scratching.

## 3. Results

### 3.1. RICTOR Demonstrated Differential Expression in Pan-Cancer

**Figure 1:**
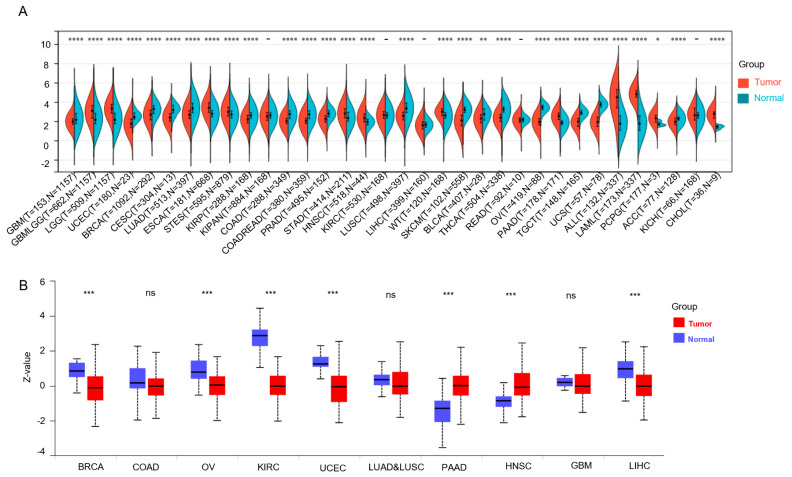
RICTOR expression profiles in cancer and normal tissues. The mRNA expression level (**A**) and the protein level (**B**) of RICTOR in pancancer data. ns, not significant, *p* > 0.05; * means *p* < 0.05; ** means *p* < 0.01; *** means *p* < 0.001; **** means *p* < 0.0001.

According to the combined tumor and normal samples from TCGA, TARGET, and GTEx, RICTOR was significantly overexpressed in GBMLGG, LGG, ESCA, STES, STAD, HNSC, WT, PAAD, ALL, LAML, PCPG, and CHOL, compared to normal tissues (Figure 1A), and significantly downregulated in GBM, UCEC, BRCA, CESC, LUAD, KIRP, COAD, COADREAD, PRAD, LUSC, SKCM, BLCA, THCA, OV, TGCT, UCS, and ACC.

Next, we extended our investigation by analyzing the protein expression levels of RICTOR using a large-scale proteome dataset obtained from the Clinical Proteomic Tumor Analysis Consortium (CPTAC) database where 10 types of cancer have protein expression data of RICTOR (Figure 1B) [10]. We found that PAAD and HNSC, which show significantly increased protein expression, also exhibit upregulated gene expression. On the other hand, BRCA, OV, KIRC, UCEC, and LIHC, show significant decreases in both protein and gene expression. This suggests a significant consistency between mRNA and protein expression in these seven types of cancer. The differential protein expression of COAD, LUAD–LUSC, and GBM are not significant, which may be attributed to posttranscriptional modifications.

### 3.2. RICTOR Has Significant Correlations with the Development and Survival in Pan-Cancer

**Figure 2:**
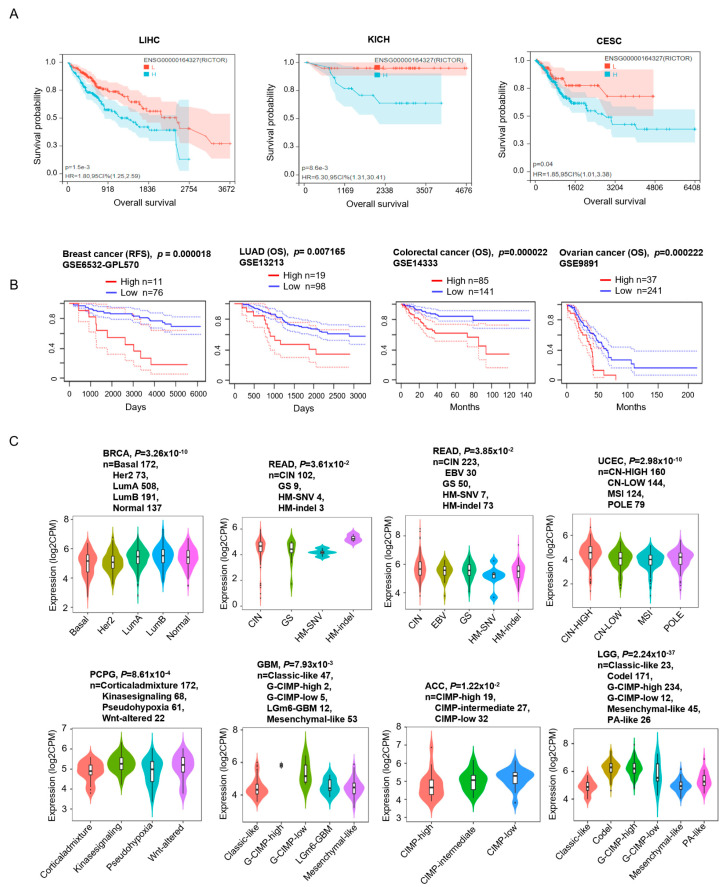
Clinicopathological feature analysis of different cancer types. Kaplan–Meier curves of cancers with significant survival differences between high and low RICTOR expression in the TCGA (**A**) and GEO dataset (**B**). (**C**) Associations between RICTOR expression and molecular subtypes. Cancers with significant differences between molecular subtypes were shown. Relapse Free Survival (RFS).

Next, we studied the association between the RICTOR expression and patient prognosis to check whether it could be used as an early diagnostic biomarker for cancers. Based on TCGA dataset and GEO dataset (from PrognoScan database), survival analysis revealed a significant correlation between higher RICTOR expression and a poorer prognosis in cases of CESC, LIHC, KICH, Breast cancer (GSE6532-GPL570), Colorectal cancer (GSE14333), Ovarian cancer (GSE9891), and LUAD (GSE13213) (Figure 2A,B). These analyses indicate that RICTOR is a valuable diagnostic biomarker in these cancer types.

Furthermore, we examined the impact of RICTOR on tumorigenesis and cell survival across different types of cancer cells using the DepMap CRISPR data. Among the 1078 cell lines, 378 cell lines’ proliferation was seriously suppressed by RICTOR gene knockout, indicating RICTOR is important for the growth and survival of these cells (Supplementary Figure S1A). By analyzing the tissue origin, Bladder Urothelial Carcinoma BC3C is the most sensitive cell line (Supplementary Table S2), and Non-Hodgkin Lymphoma is the most sensitive disease (Supplementary Table S3) for RICTOR knockout. We also find that the cancers that have a poorer prognosis in TCGA can also been found in the DepMap database, such as the cell line BOKU and CASKI of cancer CESC, with strong negative CRISPR dependency scores. Therefore, it will be meaningful to select these cancer types for further exploration of RICTOR’s regulatory mechanisms.

Next, we examined the relationship between RICTOR expression and cancer subtypes by the TISIDB database [11]. The results demonstrated a significant correlation between RICTOR expression and molecular subtypes of BRCA, GBM, ACC, LGG, PCPG, READ, STAD, and UCEC (Figure 2C). Additionally, we employed the GEPIA2 database to investigate the relationship between RICTOR expression and tumor pathological stages, and observed a significant association in SKCM, OV, and TGCT (Supplementary Figure S1B).

### 3.3. The Genetic Alteration of RICTOR Has Significant Correlations with Survival and mRNA Expression in Pan-Cancer

**Figure 3:**
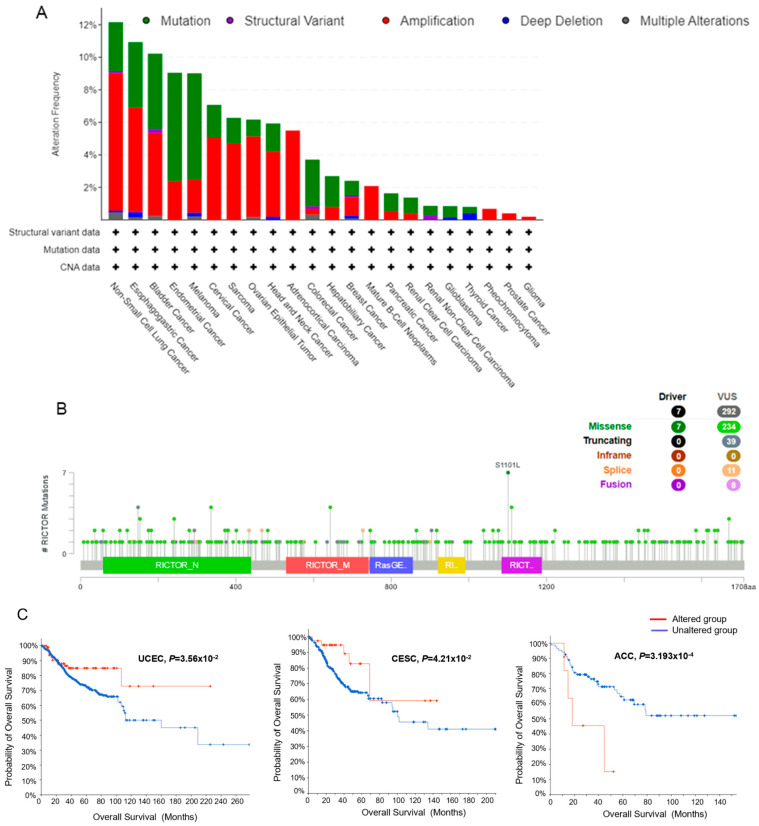

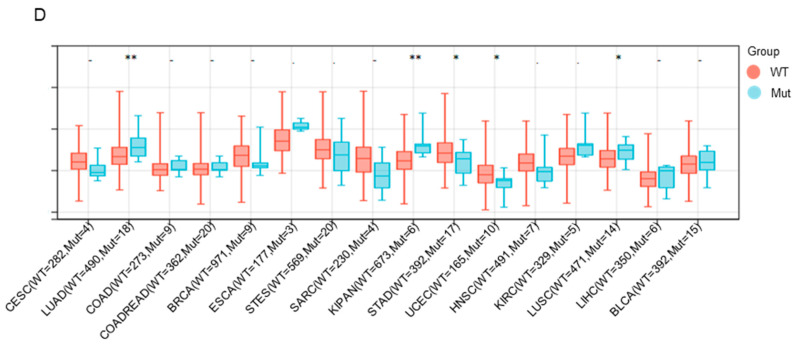
RICTOR genetic alteration analysis in pan-cancer data. (**A**) The alteration frequency of RICTOR in pan-cancer. (**B**) The mutation types of RICTOR with mutation site S1101L is visualized. VUS means variant of uncertain significance. (**C**) Kaplan–Meier curves of significant survival differences between pan-cancer data with altered RICTOR and those without altered RICTOR. Four cancers with significant survival differences were shown. (**D**) Associations between RICTOR expression and Genetic alteration. * means *p* < 0.05; ** means *p* < 0.01.

Next, we investigated the genetic alteration of RICTOR in pan-cancer. As depicted in Figure 3A, the alteration frequency of RICTOR mutations was highest in Non-Small Cell Lung Cancer (LUAD and LUSC), with a frequency of approximately 12%. Amplification was the most prevalent form of genetic alteration observed in these cancers. Esophagogastric, Bladder, Endometrial, Cervical, Sarcoma, Ovarian Epithelial, Head and Neck, and Adrenocortical Carcinoma also showed amplification-dominated variants.

Subsequently, we examined the types and sites of mutation in the RICTOR sequence. Only missense mutations were found with seven recorded samples. As a contrast, the missense variant of uncertain significance (VUS) has 234 recorded samples, which might be a potential cancer driver and in need of further investigation (Figure 3B).

We then evaluated the effect of RICTOR genetic alterations on the clinical outcomes of patients. A genetic alteration in RICTOR significantly enhanced the overall survival (OS) of patients with UCEC and CESC, as illustrated in Figure 3C. Conversely, ACC patients with RICTOR genetic alteration exhibited a significantly poor prognosis in terms of OS (Figure 3C). Nonetheless, for patients with other cancer types, RICTOR genetic alterations did not lead to a significant change in OS.

To find the possible regulatory mechanism of mutation resulting in aberrant RICTOR expression, we also examined the alteration frequency of RICTOR in pan-cancer data (Figure 3D). Our findings revealed significant correlations between RICTOR expression levels and RICTOR mutations in LUAD, KIPAN, LUSC, STAD, and UCEC. Specifically, mutations improved RICTOR expression levels in LUAD, KIPAN, and LUSC, while decreasing expression in STAD and UCEC. These results suggest that mutations may contribute to the aberrant expression of RICTOR in these cancers.

### 3.4. The Promoter Methylation Level of RICTOR Has Significant Correlations with Aberrant Expression in Pan-Cancer

**Figure 4:**
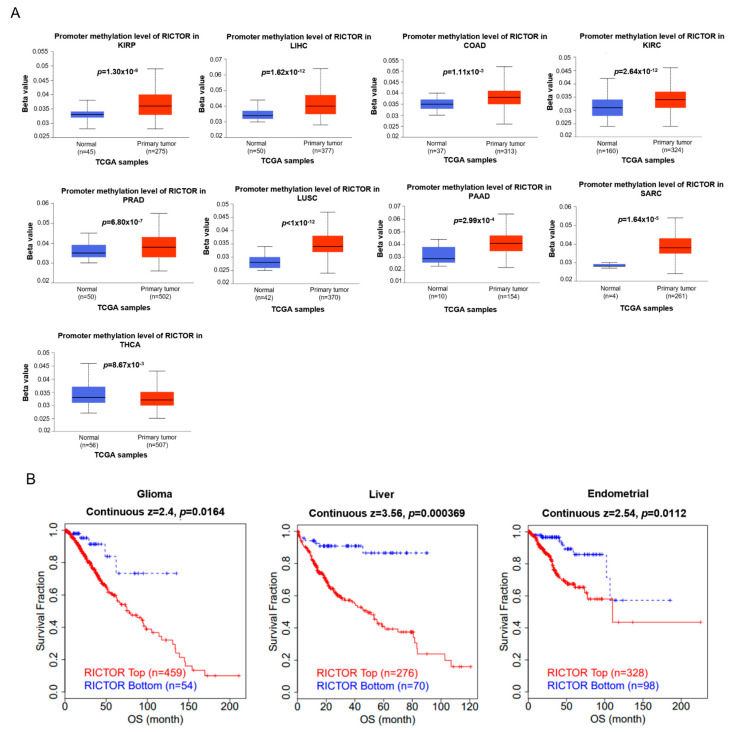
The methylation analysis of RICTOR. (**A**) The significant aberrant methylation levels of RICTOR in 9 cancers; (**B**) The significant correlation between the methylation levels of RICTOR and survival fraction in 3 cancers.

It has been demonstrated that alterations in promoter DNA-methylation patterns can impact gene expression and participate in the tumor oncogenesis [29]. Thus, we studied the methylation levels of RICTOR between normal and tumor tissues in pan-cancer data. Compared to the normal group, the promoter methylation levels of RICTOR were significantly higher in KIRP, LIHC, COAD, KIRC, PRAD, LUSC, PAAD, and SARC. Conversely, THCA exhibited significantly lower promoter methylation levels than normal tissues (Figure 4A). This finding is in line with the low RICTOR expression in KIRP, COAD, PRAD, and LUSC patients shown in Figure 1A. Further correlation analyses demonstrated that the gene expression of RICTOR was significantly negatively correlated with methylation levels in KIRP, PRAD, and LUSC (Supplementary Figure S2), indicating higher DNA methylation leads to lower gene expression in these cancers. Additionally, we did not observe significant changes in RICTOR methylation levels in other cancers. Our results indicate that an aberrant expression of RICTOR in these cancers may be attributed to the promoter methylation of RICTOR.

Next, we employed the TIDE database to analyze the correlation between methylation levels and survival outcome. As illustrated in Figure 4B, a higher methylation level of RICTOR predicted worse OS in glioma, liver, and endometrial cancer.

### 3.5. The Function of RICTOR-Interacting Genes Correlate with Cell Growth

**Figure 5:**
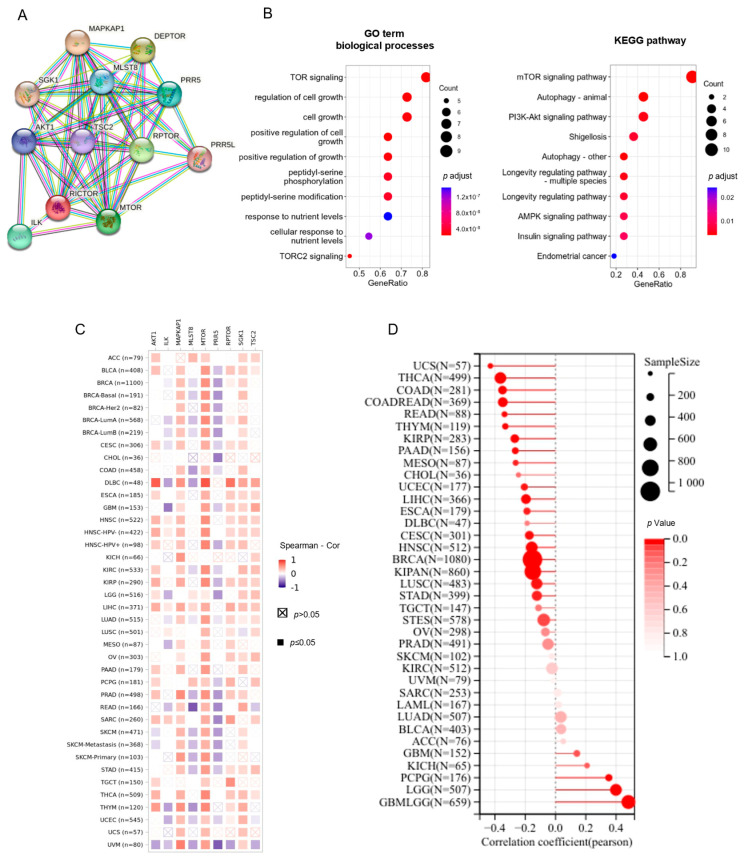
RICTOR-related gene function analysis. (**A**) RICTOR-interacting proteins network from STRING website. (**B**) GO and KEGG enrichment analyses of the top 10 interacting genes. (**C**) The heatmap of the co-expression correlation of top 10 interacting genes in pan-cancer data. (**D**) The association between the RICTOR expression and the cancer stemness score.

Next, we used functional enrichment analysis to evaluate the underlying molecular mechanisms of RICTOR in tumorigenesis and cancer development. First, we used the STRING database to find the PPI network of the RICTOR protein. The top 10 interacting proteins of RICTOR were obtained (Figure 5A). We also investigated the protein interactions in Geneamia and obtained the network, as depicted in Supplementary Figure S3A. Gene AKT1, MAPKAP1, MLST8, MTOR, PRR5 were shared in the top 10 gene lists from the two websites. Among these, MAPKAP1 is also known to be a core subunit specific to mTORC2 complex. Meanwhile, GO and KEGG enrichment analyses in Figure 5B and Figure S3B indicated that the RICTOR-interacting genes were mainly involved in TOR signaling biological processes and mTOR signaling pathway. Further pan-cancer analyses show positive correlations between the expression of RICTOR and the top 10 interacting genes in the majority of cancer types, except for gene PRR5 with negative correlation (Figure 5C and Figure S3C).

We further queried the DepMap CRISPR RICTOR knockout data for significant co-dependency Pearson correlations. Our analysis revealed that MAPKAP1 was the top co-dependency identified (Supplementary Table S4), which is consistent with their known collaborative function as core subunits that are specific to the mTORC2 complex.

### 3.6. RICTOR Has Significant Relationship with Cancer Stemness, MSI, and TMB

The initiation, development, and recurrence of cancer after chemotherapy are driven by cancer stem cells [30,31], and a greater abundance of these stem cells is linked to poor patient survival. Thus, we further investigated the correlation between RICTOR expression and the cancer stemness. Figure 5D revealed a significant positive association between the stemness of GBMLGG and RICTOR expression, with the correlation coefficient being the highest of all pan-cancers analyzed in TCGA. These results suggest that RICTOR could potentially influence the cancer stemness of gliomas and facilitate the advancement of the tumor.

TMB and MSI are biological markers spanning the entire genome, which have been utilized for forecasting the effectiveness of immune checkpoint inhibitor treatments in cancer patients. Therefore, we examined how RICTOR expression and TMB and MSI were interrelated. We observed that the level of RICTOR expression was positively linked with TMB in LUAD and SKCM, but negatively associated with TMB in UCEC, UVM, STES, and STAD (Supplementary Figure S4A). Regarding GBMLGG, we found that RICTOR expression was positively associated with MSI, whereas in DLBC, it was negatively related to MSI (Supplementary Figure S4B).

### 3.7. RICTOR Has Significant Correlations with Immune Microenvironment and Immune-Related Genes

**Figure 6:**
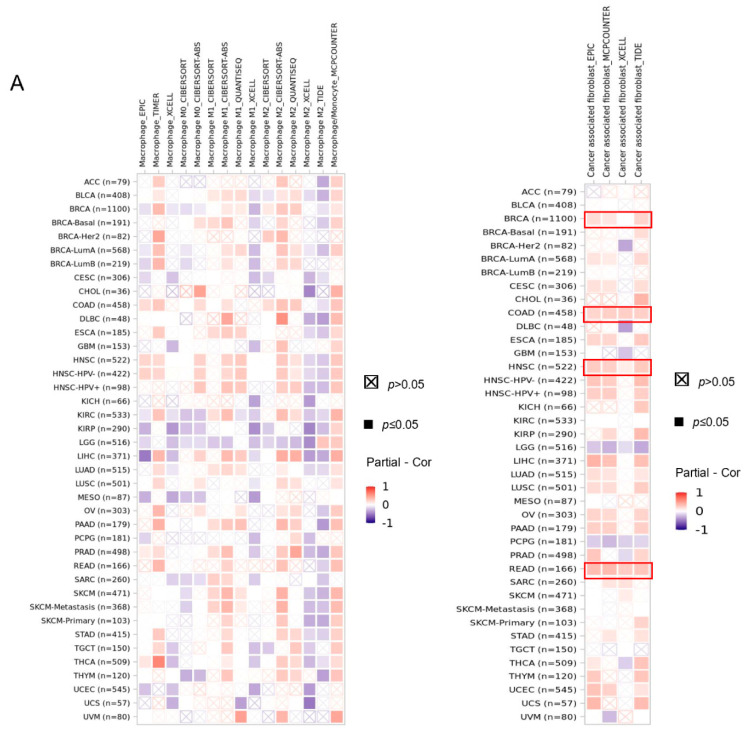

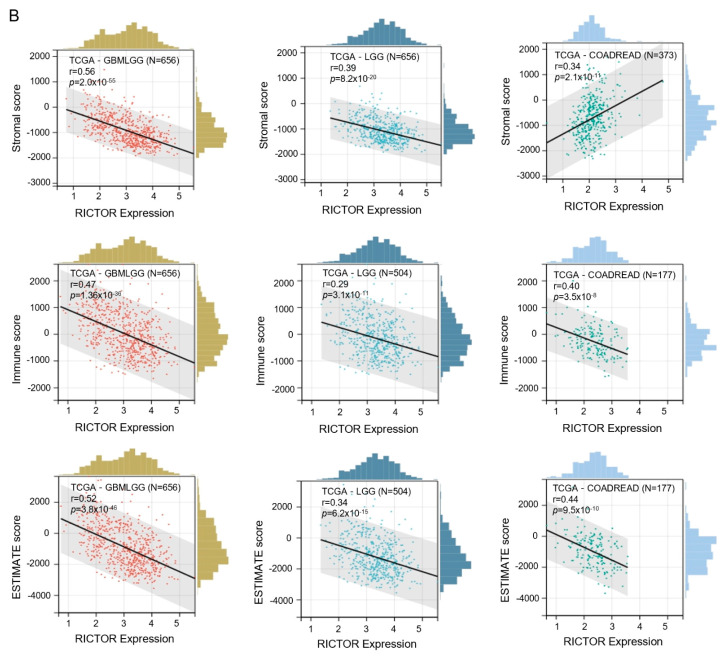
The association between the expression of RICTOR and immune microenvironment. (**A**) The correlation between the expression of RICTOR and immune cell infiltration of macrophage (**left**) and cancer-associated fibroblast (**right**) calculated by six evaluation algorithms; (**B**) The association between the RICTOR expression and Stromal score (**top**), Immune score (**middle**), and ESTIMATE score (**bottom**) with top 3 most significant correlated cancers shown.

Macrophages and CAFs are both important components of the tumor microenvironment and they play crucial roles in tumor development and metastasis [32]. Thus, we further studied whether RICTOR expression can increase or decrease the infiltration level of macrophages and CAFs (Figure 6A). We found a significantly positive association between the macrophage infiltration and RICTOR expression in COAD, HNSC, and PAAD, while KIRP, LGG, and MESO demonstrate a significantly negative correlation. In terms of CAFs, we observed a significantly positive correlation in BRCA, COAD, HNSC, and READ on all algorithms, while negative correlation in LGG and PCPG.

Infiltrating stromal and immune cells form the prominent components of normal tissue in tumor and can significantly impact cancer progression and treatment response [27]. We used the ESTIMATE method to compute the stromal, immune, and ESTIMATE scores for cancer tissue (Supplementary Table S5). We observed that RICTOR expression has most significant correlation with stromal scores in GBMLGG, LGG, and COADREAD (Figure 6B). In terms of immune scores and ESTIMATE scores, the most significant correlation was found in GBMLGG, LGG, and PCPG. Interestingly, most correlations are negative, excluding the stromal scores in COADREAD.

We further performed gene co-expression analyses to investigate the correlation between the RICTOR expression and immune-related genes in pan-cancer data, including chemokine, chemokine receptor, MHC, immunostimulator, immunoinhibitor, and immune checkpoint genes. According to the results, strong positive correlations were found between RICTOR and most of the immune-related genes in multiple cancer types except for GBMLGG and LGG which show negative correlations (Supplementary Figure S5). In detail, the expression of chemokine receptors such as CCR1, CXCR2, and CX3CR1 and chemokines such as CCL28, CXCL8, and XCL1 were positively correlated with RICTOR in various tumors. MHC genes had a co-expression with RICTOR in almost all cancer types, particularly in LIHC, ACC, BRCA, and GBM. In addition, the expression of most immunostimulatory genes and immunosuppressive genes were also positively correlated with RICTOR in TCGA pan-cancer. A high expression of immune checkpoint genes can reduce the immune response against the tumor and lead to tumor-immune escape. Thus, we explored the association between immune checkpoint genes and RICTOR (Supplementary Figure S6). Similar with immune-related genes, the transcription level of RICTOR is positively associated with most immune checkpoint genes in most cancer types except for GBMLGG and LGG. This result reveals that RICTOR may suppresses immune response by regulating immune checkpoint genes and may be a hopeful target to enhance the efficacy of immunotherapy.

### 3.8. RICTOR Knockdown Inhibits the Proliferation and Migration of Hela Cells

**Figure 7:**
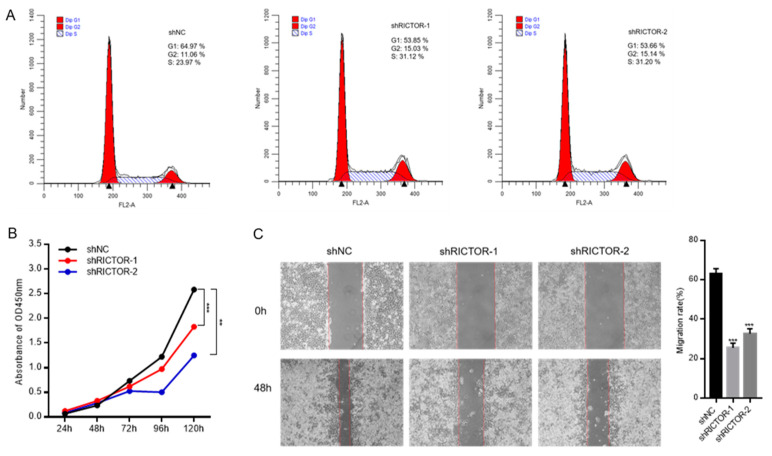
RICTOR knockdown inhibits the growth of Hela cell. (**A**) Cell cycle analysis by Flow Cytometry. (**B**) Cell proliferation assay by CCK8. (**C**) Migration rate analyzed by wound-healing assay. ** means *p* < 0.01; *** means *p* < 0.001.

To confirm the function of RICTOR in tumor growth, we selected cervical cancer cell line, Hela, to examine the effect of RICTOR knockdown on cell growth. The results demonstrated that RICTOR knockdown could induce cell cycle arrest, with a decrease in G1 phase and an increase in S and G2/M phase (Figure 7A). Additionally, the results of the CCK8 assay and wound-healing assay confirmed that cell proliferation (Figure 7B) and migration (Figure 7C) were suppressed after knockdown. Taken together, these results suggest that RICTOR may promote tumor cell growth by regulating the cell cycle.

## 4. Discussion

In this study, we performed a comprehensive analysis to investigate the molecular characteristics of RICTOR in different cancers from diverse databases, including TCGA, TARGET, CPTAC, and GEO, and the value of RICTOR in patient survival and treatment.

The PI3K/Akt/mTOR pathway plays a crucial role in cell growth, proliferation and survival, and is frequently dysregulated in cancer. AKT is phosphorylated and activated by PDK1 and mTORC2, and activated AKT then phosphorylates various downstream targets involved in cell growth and proliferation. In many cancers, including Cervical and Breast cancer, RICTOR overexpression leads to increased Akt S473 phosphorylation and promotes tumorigenesis [6,33,34].

We investigated the correlation between RICTOR expression, genetic alteration, methylation and patient prognosis to check whether they could be used as an early diagnostic biomarker for cancers. Based on our results, we found that the higher expression of RICTOR was significantly correlated with poor patient survival in multiple cancer types, including CESC, LIHC, KICH, BRCA, COAD, OV, and LUAD. On the other hand, genetic alteration has a significant correlation with the prognosis of UCEC, CESC, and ACC patients. Methylation has a significant correlation with the prognosis of GBM, LIHC, and UCEC patients. Compared with gene expression, genetic alteration and methylation have a weaker impact on prognosis.

We also observed a significant correlation between a higher expression of RICTOR and higher survival probability for LGG, SKCM, KIRC, Glioma (GSE4412-GPL97), AML (GSE12417-GPL570), Melanoma (GSE19234), and HNSC (GSE2837) (Supplementary Figure S7). This result indicates that RICTOR may act as tumor suppressor in these cancers. A high expression of RICTOR can enhance its suppressive functions, leading to better control of cell proliferation, and improved patient outcomes. Another reason is the potential involvement of RICTOR in DNA repair pathways and maintaining genomic stability. Reduced expression can compromise the cell’s ability to repair DNA damage, leading to genomic instability, accumulation of mutations, and increased susceptibility to further genetic alterations that promote cancer progression. Since the underlying mechanisms connecting gene expression to prognosis are complex and multifaceted, further research is required to fully understand its implications in different cancer contexts.

Given the upregulation in 12 cancer types, we investigated possible regulatory mechanisms of a high RICTOR expression using correlation analysis between mRNA expression, methylation and genetic alteration. We found that LUAD and LUSC have higher mRNA expression when comparing mutated and wild-type tissue, indicating mutation is one of the main reasons of RICTOR abnormal expression in lung cancer. Additionally, no significantly negative regulatory relation between methylation and mRNA expression were found, indicating that the contribution of methylation levels to the upregulation of RICTOR gene expression is negligible. As mRNA expression could be modulated at multiple levels, more regulatory mechanisms are needed to be investigated in the future.

To obtain a deep understanding of the biological function of RICTOR, we identified the top 10 interacting proteins from STRING and the Geneamia database for co-expression, GO, and KEGG pathway enrichment analysis. The enrichment analysis suggested that these genes were mainly involved in TOR signaling and cell growth, which suggested that RICTOR is essential for cell cycle regulation. These findings might provide new cues for further exploring the molecular function of RICTOR.

The immune cell plays a critical role in recognizing and eliminating cancer cells. Thus, we also checked the associations between RICTOR expression and immune microenvironment. We found a significantly positive association between the macrophage infiltration and RICTOR expression in COAD, HNSC, and PAAD. COAD and HNSC are also positively correlated with the infiltration of CAFs. According to this result, we speculate that the immunotherapy efficacy might be improved for COAD and HNSC, with RICTOR as new target, and biomarkers.

In conclusion, our pan-cancer analysis shows that RICTOR has a high expression in various cancers, and its mRNA expression, mutation, and DNA methylation are significantly associated with patient survival in certain tumors. In addition, immune microenvironment analysis and gene function analysis suggests potential mechanisms that RICTOR may regulate tumor immunity, and sustain tumor growth and proliferation. Further experimental and clinical studies are needed to confirm these findings by elucidating the molecular mechanisms underlying the relationship between RICTOR and cancer, and to explore the practical application of RICTOR in cancer therapy and prognosis prediction.

## Data Availability

The datasets used in this article are obtained from public repositories and websites. Details can be found in Materials and methods section.

## Supplementary Materials

The following supporting information can be downloaded at: https://www.mdpi.com/article/10.3390/genes14061280/s1, Figure S1: The effects of RICTOR expression on cell growth and tumor pathological stages; Figure S2: The negative correlation between gene expression and methylation level in three CpG sites in KIRP, PRAD, LUSC; Figure S3: Function analysis for RICTOR-interacting genes; Figure S4: The relationship between RICTOR expression and tumor mutation burden (TMB) (A) and microsatellite instability (MSI) (B); Figure S5: Correlation heatmap between the expression of immune-related genes and RICTOR in pan-cancer data; Figure S6: Correlation heatmap between the expression of immune checkpoint genes and RICTOR in pan-cancer data; Figure S7: Kaplan-Meier curves of cancers with significant survival differences between high and low RICTOR expression in TCGA (A) and GEO dataset (B); Table S1: TCGA and TARGET datasets used in this study; Table S2: Top cell lines most sensitive to RICTOR knockout by CRISPR/Cas9; Table S3: The frequency at which the Primary.Disease has been recorded; Table S4: RICTOR’s Top 100 Codependencies with Pearson correlations from DepMap; Table S5: RICTOR expression and immune infiltration scores in pan-cancer.
