# Supplementary material for: A Comprehensive Pan-Cancer Analysis of the Potential Biological Functions and Prognosis Values of RICTOR
Source: Genes (Basel). 2023 Jun 16;14(6):1280. doi: 10.3390/genes14061280 (PMC10298440; doi:10.3390/genes14061280)
Supplement: Supplementary file 1 [file genes-14-01280-s001.zip › supplementary_figs.pdf]

Supplementary Materials

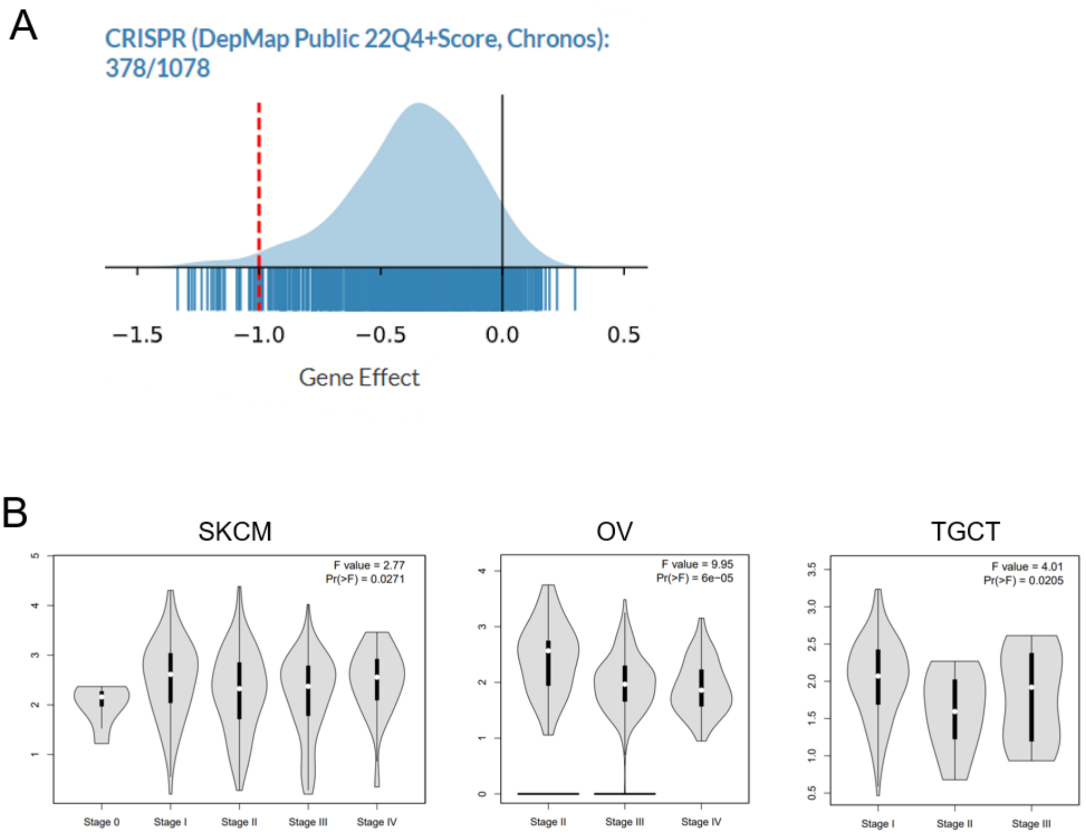

Figure S1. (A) Schematic representation of the effects of RICTOR gene knockout by CRISPR on cell growth and survival. X-axis: dependency scores (Gene Effect) reflect the dependence of cell growth and survival upon depletion of a particular gene. Negative values indicate that cell proliferation is decreasing upon the gene depletion and score less than −0.5 indicates that the gene is essential for survival of a given cancer cell line. Numbers next to "CRISPR" indicate the amount of sensitive cell lines with score <−0.5/total number of cell lines. (B) Relationship between RICTOR expression levels and tumor pathological stages in different cancer performed in GEPIA2. Cancers with the significant F-values were shown.

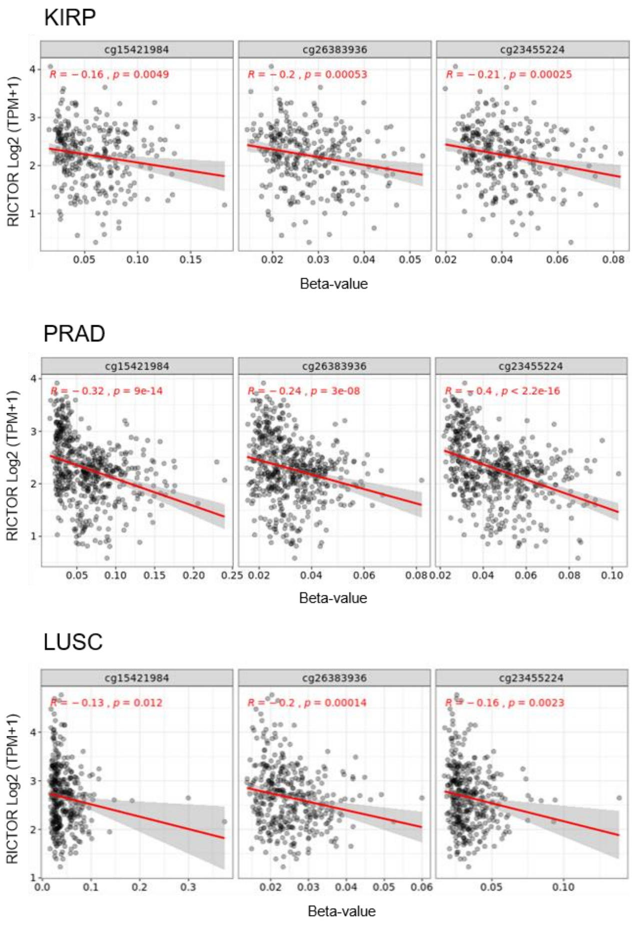

Figure S2. The negative correlation between gene expression and methylation level in three CpG sites in KIRP, PRAD, LUSC

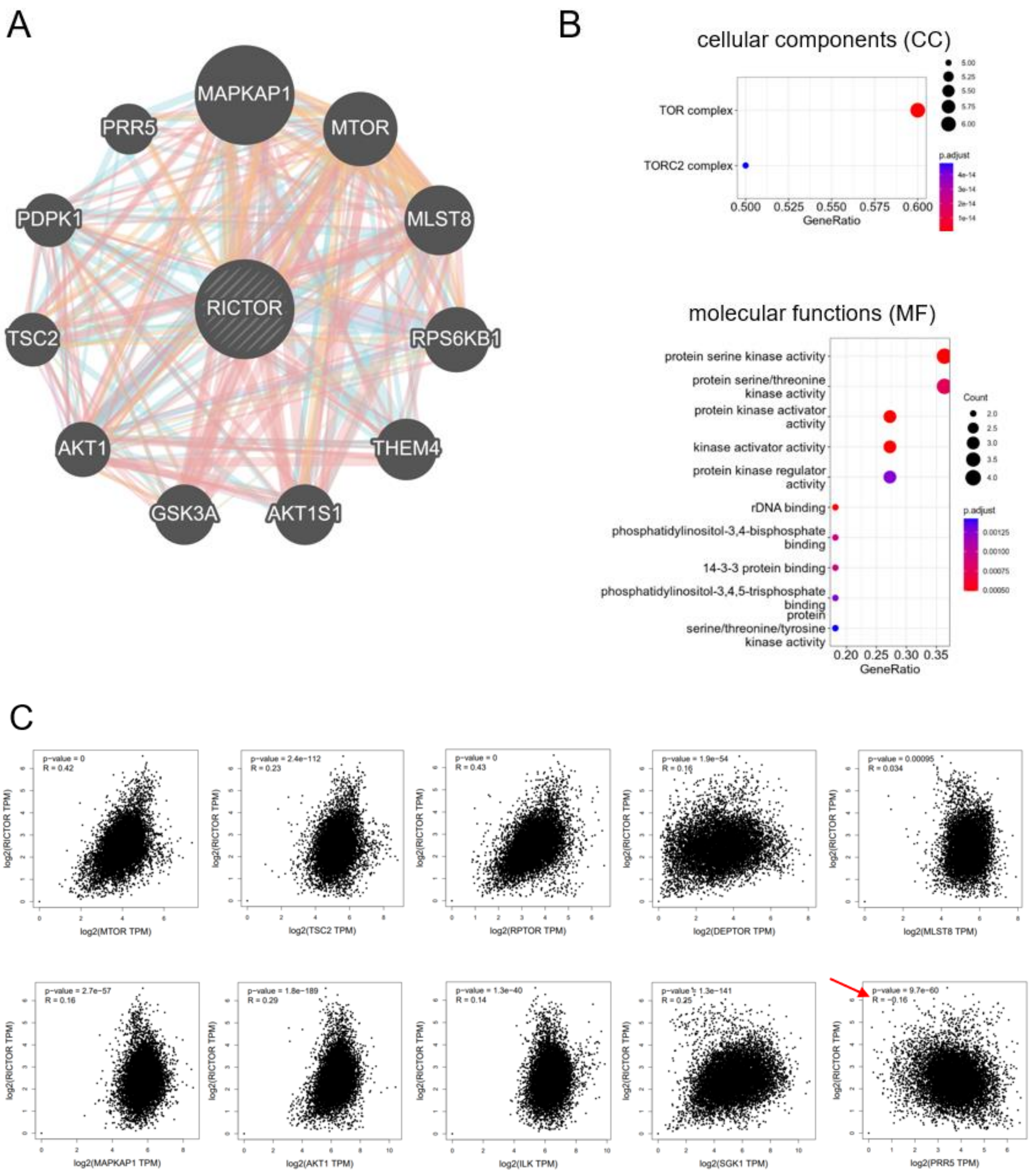

Figure S3. RICTOR-related gene function analysis. (A) RICTOR-interacting proteins network from Geneaminia website. (B) GO analyses (cellular components and molecular functions) of the top 10 interacting genes. (C) Correlation analysis between RICTOR and top 10 interacting genes conducted by GEPIA2 across all tumor samples from TCGA.

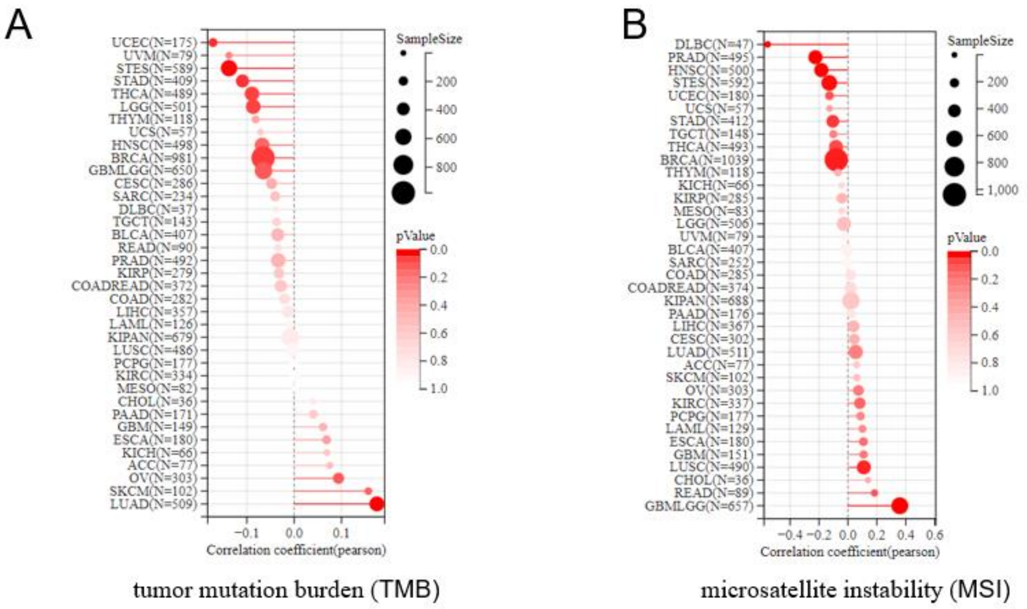

Figure S4. The relationship between RICTOR expression and tumor mutation burden (TMB) (A) and microsatellite instability (MSI) (B).

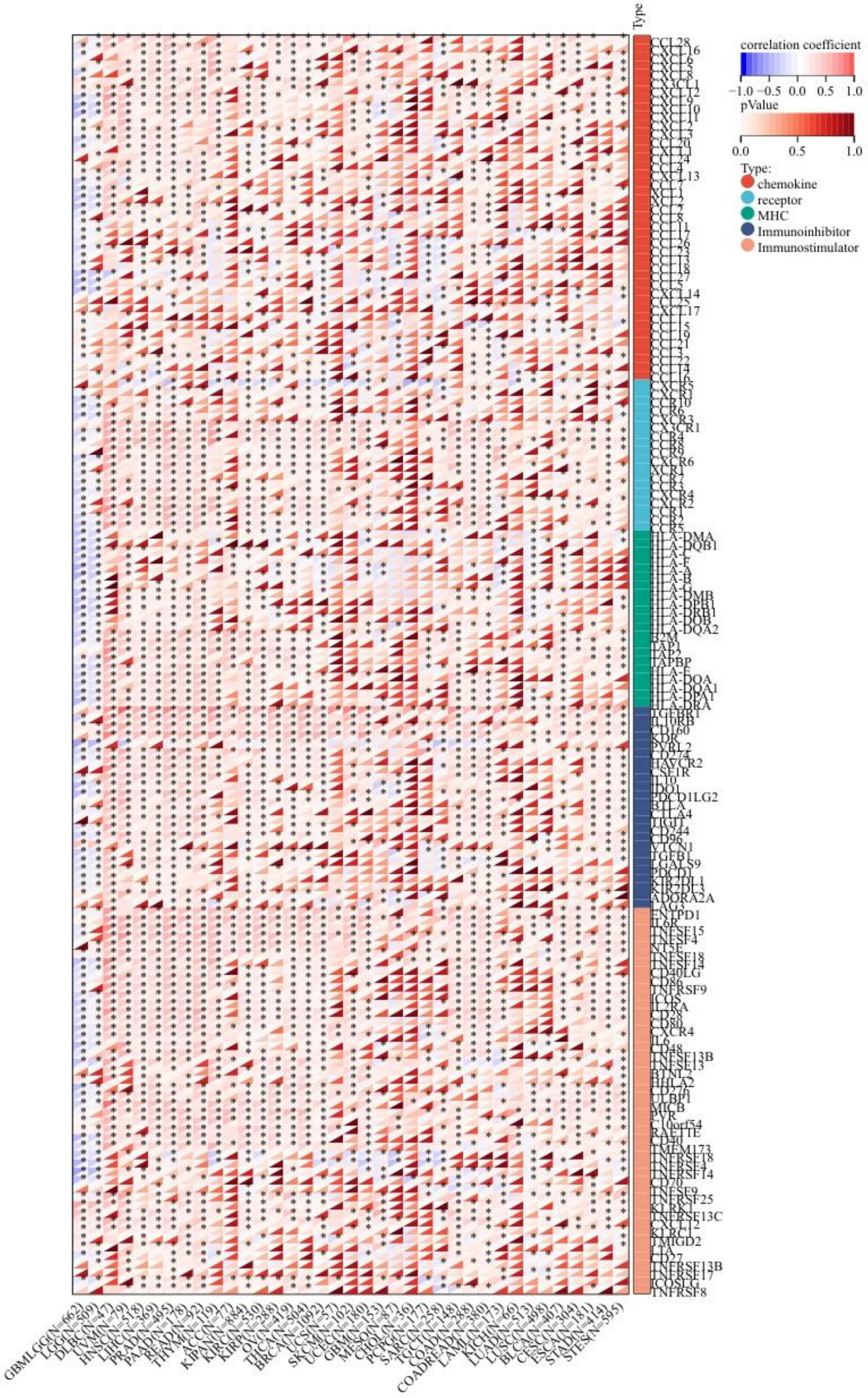

Figure S5. Correlation heatmap between the expression of immune-related genes and RICTOR in pan-cancer data. * means statistically significant.

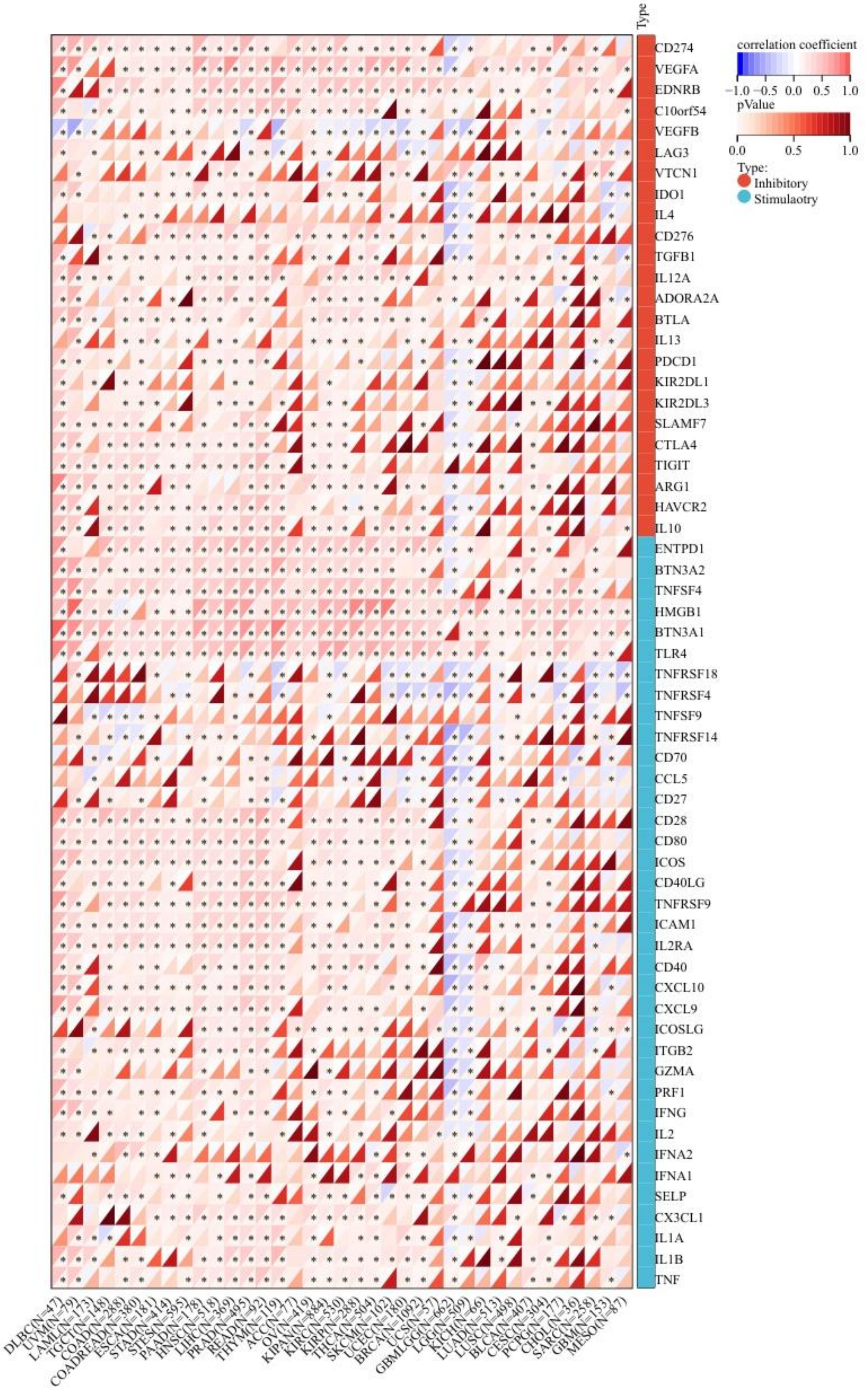

Figure S6. Correlation heatmap between the expression of immune checkpoint genes and RICTOR in pan-cancer data. * means statistically significant.

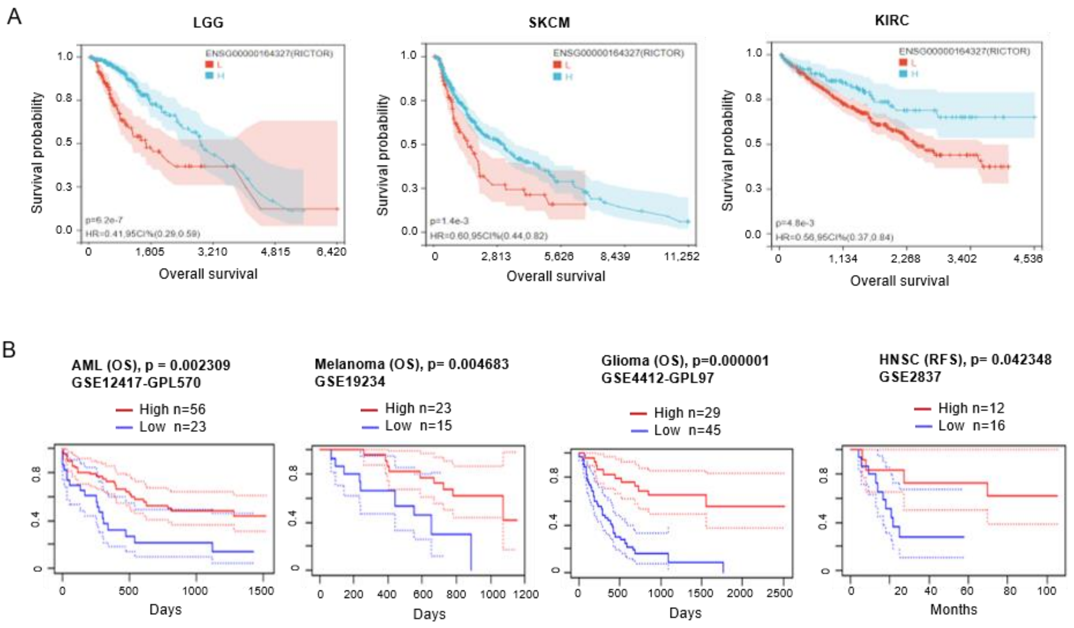

Figure S7. Kaplan-Meier curves of cancers with significant survival differences between high and low RICTOR expression in TCGA (A) and GEO dataset (B).
